# Coordinated Regulation of Protoperithecium Development by MAP Kinases MAK-1 and MAK-2 in *Neurospora crassa*

**DOI:** 10.3389/fmicb.2021.769615

**Published:** 2021-11-26

**Authors:** Nan Lan, Shuting Ye, Chengcheng Hu, Zhiling Chen, Jun Huang, Wei Xue, Shaojie Li, Xianyun Sun

**Affiliations:** ^1^Institute of Microbiology, Chinese Academy of Sciences, Beijing, China; ^2^College of Life Sciences, University of Chinese Academy of Sciences, Beijing, China; ^3^College of Life Sciences, Capital Normal University, Beijing, China; ^4^Shandong Jinniu Group Company, Ltd., Jinan, China

**Keywords:** MAP kinase, sexual development, protoperithecium, MAK-1, MAK-2, *Neurospora crassa*

## Abstract

Mitogen-activated protein (MAP) kinase pathways function as signaling hubs that are integral for many essential cellular processes, including sexual development. The molecular mechanisms and cross-talk between PR and CWI MAP kinase pathways have been extensively studied during asexual development. However, if these can be extended to sexual development remains elusive. By analyzing genome-wide transcriptional responses to deletion of each of two MAP kinase coding genes *mak*-*2* (PR-MAP kinase pathway) and *mak*-*1* (CWI-MAP kinase pathway) in *Neurospora crassa* during protoperithecium formation, 430 genes co-regulated by the MAK-1 and MAK-2 proteins were found, functionally enriched at integral components of membrane and oxidoreductase. These genes include 13 functionally known genes participating in sexual development (*app*, *poi-2*, *stk-17*, *fsd-1*, *vsd-8*, and NCU03863) and melanin synthesis (*per-1*, *pkh-1*, *pkh-2*, *mld-1*, *scy-1*, *trn-2*, and *trn-1*), as well as a set of functionally unknown genes. Phenotypic analysis of deletion mutants for the functionally unknown genes revealed that 12 genes were essential for female fertility. Among them, single-gene deletion mutants for NCU07743 (named as *pfd-1*), NCU02250 (*oli*), and NCU05948 (named as *pfd-2*) displayed similar protoperithecium development defects as the Δ*mak*-*1* and Δ*mak*-*2* mutants, failing to form protoperithecium. Western blotting analysis showed that both phosphorylated and total MAK-1 proteins were virtually abolished in the Δ*nrc*-*1*, Δ*mek*-*2*, and Δ*mak*-*2* mutants, suggesting that the posttranscriptional regulation of MAK-1 is dependent on the PR-MAP kinase pathway during the protoperithecium development. Taken together, this study revealed the regulatory roles and cross-talk between PR and CWI-MAP kinase pathways during protoperithecium development.

## Introduction

Sexual reproduction is common in the eukaryotes and is a quick way for generating genetic diversity in offspring. In multicellular filamentous fungi, it is a complex process involving the development of sexual reproductive structures (Ni et al., 2011; Wilson et al., 2021). *Neurospora crassa* is a multicellular ascomycete fungus in the family Sordariaceae and has long been used as an excellent model organism for genetic and biochemical research and the study of sexual development—the fruiting body morphogenesis (Perkins and Davis, 2000; Davis and Perkins, 2002). *N*. *crassa* is heterothallic with two mating types, designated as *mat a* and *mat A*. Both mating types can form a protoperithcium, the female sexual reproductive organ, and then develop into perithecium after fusing the nuclei from two opposite mating types (Nelson and Metzenberg, 1992). In *N*. *crassa* and other members of Sordariaceae, the entire process of sexual development goes through three main stages, from ascogonial, protoperithecial, to perithecial stages (Lord and Read, 2011; Lichius et al., 2012). The ascogonial stage includes ascogonial coil initiation, coil extension, coil adhesion, septation, and branching. The protoperithecial stage includes enveloping hyphal growth, hyphal adhesion, extracellular matrix deposition, septation and branching, conglutinate cell formation, and trichogyne formation. The perithecial stage begins after nuclear fusion and is marked by dikaryon formation. Ascospores are then generated after meiosis in the formed perithecium. The entire process undergoes hyphal aggregation, adhesion, septation, branching, and cell differentiation involving at least 14 distinct cell types (Lichius et al., 2012). Therefore, perithecium morphogenesis in *N*. *crassa* also provides an ideal model system for studying the development of multicellular organisms.

Signal transduction pathways play key roles in fruiting-body morphogenesis. Some of the most important pathways, the mitogen-activated protein (MAP) kinase pathways, are highly conserved and well-characterized in eukaryotes (Chen and Thorner, 2007; Krishna and Narang, 2008). Three-tiered MAP kinase cascades have been identified in *N*. *crassa*, including the pheromone response (PR) pathway, the cell wall integrity (CWI) pathway, and the osmoregulatory (OS) pathway, which are involved in the regulation of pheromone-induced mating and filamentous growth, cell wall modification and repair, and responses to high osmolarity, respectively (Paul et al., 1997; Posas et al., 1998; Lengeler et al., 2000; Bahn et al., 2007; Chen and Thorner, 2007; Saito, 2010). In each of the pathways, the signal from external or internal is transduced from MAP kinase kinase kinase (MAPKKK) to MAP kinase kinase (MAPKK) and then to MAP kinase; the MAP kinase phosphorylate downstream targets to exert cellular responses to the signal. In the PR-MAP kinase pathway, the three kinases are NRC-1, MEK-2, and MAK-2; in the CWI MAP kinase pathway, they are MIK-1, MEK-1, and MAK-1; whereas, in the OS MAP kinase pathway, they are OS-5, OS-4, and OS-2. Previous studies have shown that mutants lacking any kinase of the MAP kinase pathways were unable to produce fertilizable protoperithecia in *N. crassa* (Pandey et al., 2004; Li et al., 2005; Maerz et al., 2008; Park et al., 2008; Chinnici et al., 2014). Lichius et al. (2012) performed an in-depth comparison of the protoperithecium morphogenesis in these mutants and wild type by low-temperature scanning electron microscope and showed that three MAP kinase cascades each have distinctly different functions during sexual development in *N. crassa*. In addition to *N*. *crassa*, deletion mutants missing genes in the MAP kinase cascades also showed defects in fruiting body development in several other fungal species, such as *Aspergillus nidulans* (Wei et al., 2003), *Fusarium graminearum* (Hou et al., 2002), *Magnaporthe grisea* (Xu et al., 1998), and *Cochliobolus heterosporus* (Lev et al., 2009), indicating their conserved roles in sexual development.

For PR- and CWI-MAP kinase pathways, their roles in cell fusion, asexual development, and cell wall integrity have been extensively studied in filamentous fungi by combined genetic, biochemical, and transcriptomic data (Bennett et al., 2013; Fischer et al., 2018; Fischer and Glass, 2019). A very interesting phenomenon is that several studies have documented cross-talk between the MAK-1 and MAK-2 pathways in *N. crassa* and in other filamentous ascomycete fungi in the asexual development stage (Maerz et al., 2008; Dettmann et al., 2012, 2013; Maddi et al., 2012; Leeder et al., 2013; Fu et al., 2014; Fischer and Glass, 2019). However, the underlying genetic basis and regulatory mechanism of these two MAP kinase pathways in multicellular sexual development remain elusive, and if their cross-talk can be extended to sexual development is unknown.

In this study, we performed a comparative genome-wide transcriptional analysis in the early stage of the protoperithecial morphogenesis in the Δ*mak-2* mutant of the PR-MAP kinase pathway, the Δ*mak-1* mutant of the CWI-MAP kinase pathway, and wild type of *N. crassa* to unveil the systematic and comprehensive genetic basis of protoperithecium development regulated by these MAP kinase pathways, and cross-talk between PR- and CWI-MAP kinase pathways in these complicated and coordinated sexual developmental processes.

## Materials and Methods

### Strains and Culture Conditions

All *N*. *crassa* strains used in this study, including Fungal Genetics Stock Center (FGSC)#4200 (wild type, a), FGSC#2225 (wild type, A), FGSC#11482 (Δ*mak-2*, a), FGSC#11321 (Δ*mak-1*, a), and knockout mutants for phenotypic analysis were obtained from the Fungal Genetics Stock Center^1^ (University of Kansas Medical Center). Strains were maintained on Vogel’s slant (1× Vogel’s salts, 2% sucrose, and 1.5% Bacto Agar), Vogel’s plate (1× Vogel’s salts, 2% glucose, and 0.75% Bacto Agar), and liquid Vogel’s medium (1× Vogel’s salts, and 2% glucose). Synthetic crossing (SC) medium with 0.1% sucrose and 0.025 SC medium with 2% sucrose were used for protoperithecial production and evaluation, respectively (Westergaard and Mitchell, 1947). Strains were grown at 28°C for vegetative growth and asexual sporulation and at 25°C for sexual development.

### Sample Preparation for RNA Sequencing and Quantitative Reverse Transcription-Polymerase Chain Reaction Analysis

Genome-wide transcriptional profiles of the Δ*mak-1* mutant, the Δ*mak-2* mutant, and the wild-type strain at the vegetative growth stage and the initial stage of protoperithecium formation were obtained by RNA sequencing. To obtain RNA at the vegetative growth stage, strains were cultured in liquid Vogel’s medium at 28°C for 22 h with orbital shaking, and the mycelia were harvested by vacuum filtration. To obtain RNA in the initial stage of protoperithecium formation, strains were inoculated on a solid synthetic crossing medium with 0.1% sucrose, on which sterilized cellophane was overlaid. Plates were incubated under constant dark at 25°C for 3.5 and 5.5 days.

Mycelia were immediately frozen after harvest and ground into fine powder in liquid nitrogen. Total RNA was extracted according to the standard TRIzol protocol (Invitrogen Corporation, Carlsbad, CA, United States). For transcriptomic profiling analysis, mixed RNA from three repeats were sent to Beijing Genomics Institute for RNA-seq analysis using the Illumina Hiseq2000 with a single-end module (Illumina, San Diego, CA, United States). The raw data have been deposited in the National Center for Biotechnology Information with the accession number GSE184024. The data analysis followed the method described by Sun et al. (2019). In this study, genes with a transcriptional change for more than twofold between two samples and genes with reads per kilobase of exon model per million mapped reads value of more than 10 in at least one sample were defined as differentially expressed genes.

For quantitative reverse transcription-polymerase chain reaction (RT-qPCR) analysis, RNA extraction, DNase I treatment, complementary DNA synthesis, and qPCR analysis followed the methods described previously (Sun et al., 2019). Each complementary DNA sample was analyzed in triplicate, and the average threshold cycle was calculated. Relative expression levels were calculated using the 2^–ΔΔ*Ct*^ method and normalized to the expression of β-*tubulin* (Livak and Schmittgen, 2001). Primer pairs used for RT-qPCR assay are shown in Supplementary Table 1.

### Assessing Phenotypes of Gene Deletion Mutants

Protoperithecium formation in knockout mutants for candidate genes co-regulated by the MAK-1 and MAK-2 proteins was observed, and mutants defective in protoperithecium formation or female fertility were screened. First, the wild-type and single-gene deletion mutants were individually inoculated on Vogel’s slants and grown at 28°C for 7 days. The fresh conidia were then harvested into distilled water, and the final concentration of conidial suspensions was adjusted to 10^7^ conidia/ml. Three microliters of the conidial suspension were inoculated on cellophane covered on an SC plate with 0.1% sucrose and cultured at 25°C. After 3.5, 5.5, and 7.5 days, protoperithecium was observed under a microscope.

To evaluate the female fertility of mutants, strains were inoculated onto an autoclaved filter paper covered on a sugar-free SC medium plate and incubated at 25°C in constant darkness for 5 days. Then, the conidial suspension of wild type with opposite mating type, a (#4200) or A (#2225), was inoculated on the colony of the female strain and cultured in constant darkness for 7 days, followed by continuous illumination for more than 1 week. Perithecium formation and ascospore ejection were observed by microscope.

As many of the isolates in the *N. crassa* single-gene deletion library have mutations in addition to the targeted deletion (Fu et al., 2011), these mutants with defects in protoperithecium or perithecium formation were subjected to check if their phenotype co-segregate with hygromycin resistance according to the co-segregation database constructed by Stephen Free’s group^2^ (Chinnici et al., 2014). Also, the one that did not co-segregate was removed from the final data.

### Protein Extraction and Western Blotting

The sample preparation for protein extraction was the same as RNA-seq. After harvest, the mycelium of each sample was ground in liquid nitrogen and mixed with the proper amount of protein extraction buffer [50-mM 4-(2-hydroxyethyl)-1-piperazineethanesulfonic acid, pH 7.4; 137-mM sodium chloride; 10% glycerol] with phosphatase inhibitor mixture (All-in-One, APPLYGEN, China). After centrifugation (4°C, 12,000 rpm, 30 min), the supernatants were transferred to new Eppendorf tubes and centrifuged again if necessary. The protein concentration was determined using a Nanodrop spectrophotometer (BioSpec-nano, SHIMADZU Biotech). A proper volume of protein extracts with 30-μg total protein per lane was applied to sodium dodecyl sulfate-polyacrylamide gel electrophoresis and subsequent Western blotting. To analyze the phosphorylation status of the MAK-1 and MAK-2 proteins, a polyclonal rabbit phospho-p44/42 MAP kinase antiserum (Cell Signaling Technology, United States) was used as a primary antibody, and AffiniPure goat anti-rabbit immunoglobulin G (IgG) (H+L) (APPLYGEN) was used as the secondary antibody (Roux and Blenis, 2004; Baccarini, 2005). The polyclonal rabbit Kss1 antiserum (y-50) (Santa Cruz Biotechnology, Inc.) (Chapman and Asthagiri, 2009), polyclonal goat Fus3 antiserum (yC-19) (Santa Cruz Biotechnology, Inc.) (Rodríguez-Escudero et al., 2006), and anti-β-tubulin mouse monoclonal antibody (CWBIO) were used as primary antibody to detect protein levels of MAK-1, MAK-2, and β-tubulin, respectively. AffiniPure goat anti-rabbit IgG (H+L) (APPLYGEN), horse anti-goat (H+L) (Dogesce), and goat anti-mouse IgG (H+L) (Multisciences) were used as secondary antibody.

## Results

### MAK-1 and MAK-2 Are Critical for Sexual Development

All three MAP kinase cascades are required for growth and development in *N*. *crassa* (Lichius et al., 2012). More interestingly, deletion mutants of genes *mak*-*2* and *mak-1*, which, respectively, encode the most downstream kinases of PR- and CWI-MAP kinase pathways, shared some phenotypic similarities. As shown in Supplementary Figure 1A, when grown in Vogel’s agar tubes, both the Δ*mak*-*2* and Δ*mak-1* mutants produced short aerial hyphae and fewer conidia, as compared with wild type. On synthetic crossing medium, *N. crassa* wild type formed regular black subspherical protoperithecia with trichogyne. However, only several abnormal mycelial knots were found on the colony surface of the Δ*mak-1* and Δ*mak-2* mutants (Supplementary Figure 1B). After mating, protoperithecia in the wild-type strain further developed into perithecium, whereas no similar structures were found in either the Δ*mak-1* mutant or the Δ*mak-2* mutant (Supplementary Figure 1C). These results are consistent with a previous study (Pandey et al., 2004). Phenotypic similarities in hyphal growth and asexual and sexual development between the Δ*mak-1* and Δ*mak-2* mutants suggest that PR- and CWI-MAP kinase pathways have some functional overlap.

However, these two mutants also displayed obvious differences in colony growth. The Δ*mak*-*1* mutant formed a rosette-like colony, which was not seen in the Δ*mak*-*2* mutant, and its colony growth was much slower than that of the Δ*mak*-*2* mutant (Supplementary Figure 1A; Pandey et al., 2004; Li et al., 2005; Maerz et al., 2008), suggesting that the MAK-1 and MAK-2 proteins might involve in similar biological processes but have some different functions.

### Genome-Wide Transcriptional Responses to Single-Gene Deletion of *mak-1* and *mak-2*

**FIGURE 1:**
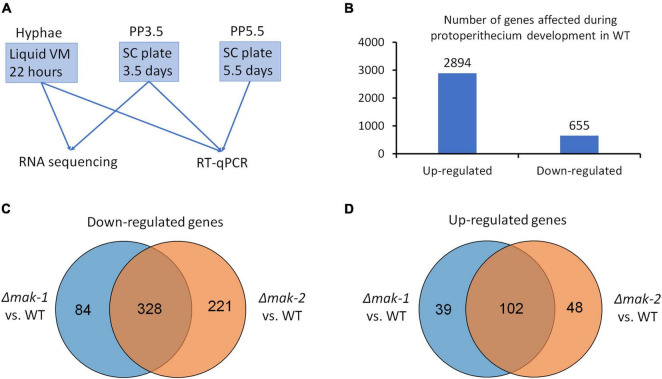
Genes responsive to sexual development induction are regulated by MAK-1 and MAK-2 proteins. **(A)** A schematic representation of sample preparation used for RNA sequencing and RT-qPCR. **(B)** Number of genes whose expressions were up- and downregulated during protoperithecium development (PP3.5) in wild type. **(C)** Venn analysis of genes downregulated in Δ*mak-1* and Δ*mak-2* mutants. Genes selected are among 2,894 genes that were upregulated in protoperithecia compared with hyphae in wild type, but their upregulation was attenuated in Δ*mak-1* and Δ*mak-2* mutants. **(D)** Venn analysis of genes upregulated in Δ*mak-1* and Δ*mak-2* mutants. Genes selected are among 655 genes that were downregulated in protoperithecia compared with hyphae in wild type, but their downregulation was weakened in Δ*mak-1* and Δ*mak-2* mutants.

To gain an insight into the molecular mechanisms of the MAK-1 and MAK-2 proteins on the regulation of protoperithecium development of *N. crassa*, we examined genome-wide transcriptional responses to single-gene deletion of *mak-1* and *mak-2*. Samples of wild type, the Δ*mak-1* and Δ*mak-2* mutants from two developmental stages, including the early stage of protoperithecium formation (3.5 days on SC medium) and the vegetative growth stage, were used for RNA sequencing (Figure 1A). A total of 9,873 genes were mapped onto the genome. In wild type, 2,894 genes were upregulated, and 655 genes were downregulated in the early stage of protoperithecium formation as compared with vegetative growth stage (Figure 1B, Supplementary Data 1, and Supplementary Table 2). These results indicate that *N. crassa* has a genome-wide transcriptional response to the induction of protoperithecium development.

The RNA-seq results also revealed that the transcriptional responses of some genes were affected by the deletion of the *mak-1* or *mak-2* gene. Among the 2,892 upregulated genes during the protoperithecium formation in wild type, the transcript levels of 412 genes in the Δ*mak-1* mutant and 549 genes in the Δ*mak*-2 mutant were dramatically lower than those in wild type (Figure 1C). Among the 622 downregulated genes during the protoperithecium formation in wild type, transcript levels of 141 genes in the Δ*mak-1* mutant and 150 genes in the Δ*mak*-2 mutant were dramatically higher than those in wild type (Figure 1D). Interestingly, 328 genes were downregulated in both the Δ*mak-1* and Δ*mak-2* mutants during protoperithecium formation, and they account for 79.6 and 59.7% of genes whose expressions were downregulated in the Δ*mak-1* and Δ*mak-2* mutants (Figure 1C and Supplementary Data 2). Also, 102 genes were upregulated in both the Δ*mak-1* and Δ*mak-2* mutants, accounting for 72.3 and 68.0% of genes whose expression was upregulated in the Δ*mak-1* and Δ*mak-2* mutants, respectively (Figure 1D and Supplementary Data 2). These results indicate that there is a large overlap in the regulatory functions between the MAK-1 and MAK-2 proteins. GO enrichment analysis revealed that, among the 430 genes affected by both the *mak-1* and *mak-2* gene deletions, some are related to oxidoreductase activity, whereas some encode proteins belonging to integral components of a membrane (*p* < 0.01) (Supplementary Table 3).

**FIGURE 2:**
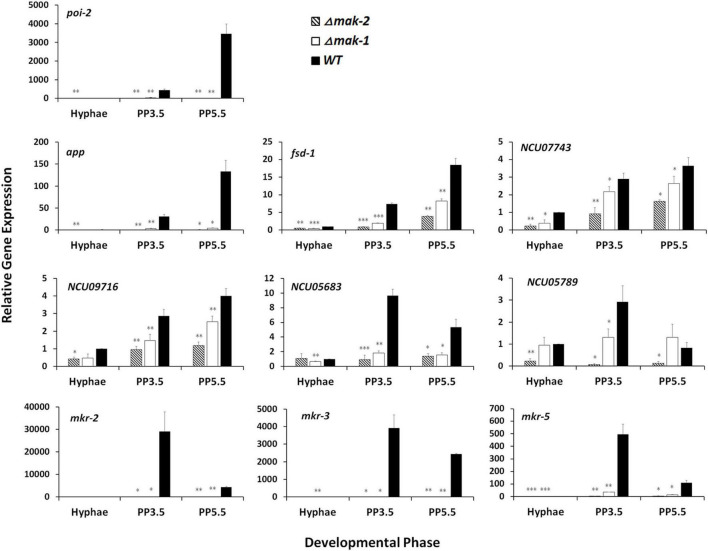
Confirmation of impacts of *mak-1* and *mak-2* in transcriptional responses by selected genes to sexual development induction through RT-qPCR analysis. Hyphae: samples of *N. crassa* in vegetative growth stage; PP3.5: samples of *N. crassa* after 3.5 days of sexual development induction; PP5.5: samples of *N. crassa* after 5.5 days of sexual development induction. Relative gene expression in Δ*mak-1* and Δ*mak-2* mutants vs. wild type (WT) was calculated using 2^– ΔΔ*CT*^ method. All expression levels were normalized to expression level of β-tubulin. Bars represent mean and standard error for three replicates. Analysis of variance statistically analyzed differences between mutants and wild type. Values with *P* < 0.001, 0.001 < *P* < 0.01, and 0.01 < *P* < 0.05 are marked with ^***^, ^**^, and *, respectively.

Among these genes, *mkr*-*2*, *mkr*-*3*, and *mkr*-*5*, three downstream genes in the PR-MAP kinase pathway, are specifically expressed during sexual development (Li et al., 2005). RT-qPCR further verified the regulation of these three genes by MAK-1 and MAK-2. As shown in Figure 2, the transcript levels of these genes dramatically increased during the protoperithecium formation and peaked after growth for 3.5 days in wild type. However, in both the Δ*mak*-*2* mutant and the Δ*mak*-*1* mutant, the transcript levels of *mkr*-*2*, *mkr*-*3*, and *mkr*-*5* were dramatically lower than those in wild type, and the transcript levels of *mkr*-*2* and *mkr*-*3* were almost undetectable (Figure 2).

In addition, some sexual development essential genes, such as *poi-2*, *app*, and *fsd-1*, and several genes with unknown functions, such as *NCU07743*, *NCU09716*, *NCU05683*, and *NCU05789*, were also selected for RT-qPCR analysis. In wild type, the transcript levels of these genes were dramatically increased during protoperithecium formation, but these increases were abolished or greatly reduced in both the Δ*mak-1* and Δ*mak-2* mutants (Figure 2). All these results demonstrate that protoperithecium development is co-regulated by the MAK-1 and MAK-2 proteins.

To examine whether these genes are regulated by the known transcription factors (ADV-1 and PP-1) downstream of MAK-1 and MAK-2 proteins, we analyzed the ADV-1 and PP-1 binding sites of these co-regulated genes in their promoters. Among the 430 genes co-regulated by the MAK-1 and MAK-2 proteins during protoperithecium development, a total of 167 genes have ADV-1 and/or PP-1 binding sites in their promoters, including several transcription factors (Supplementary Data 2). In addition, among the 430 co-regulated genes, 26 were regulated by ADV-1 and/or PP-1 during hyphal growth (Fischer et al., 2018). These results indicate that the MAK-1 and MAK-2 proteins regulated protoperithecium development may be partially through ADV-1 and/or PP-1, and there may be other transcription factors downstream of the MAK-1 and MAK-2 proteins involved in protoperithecium development.

### Known Genes Participating in Sexual Development Are Positively Co-regulated by the MAK-1 and MAK-2 Proteins

Among the 430 genes co-regulated by the MAK-1 and MAK-2 proteins in the early stage of protoperithecium development, roles of 61 genes in sexual development were previously assayed (Kim and Nelson, 2005; Nowrousian et al., 2007; Fu et al., 2011; Park et al., 2011; Chinnici et al., 2014; Carrillo et al., 2017; Fischer and Glass, 2019), including *app*, *poi-2*, *stk-17*, *fsd-1*, *vsd-8*, and NCU03868 (Table 1 and Supplementary Data 2). The gene *app* (abundant perithecial protein) is an indicator of sexual development, and its transcripts can be detected after the onset of protoperithecium development (Nowrousian et al., 2007). The remaining five genes, *poi-2*, *stk-17*, *fsd-1*, *vsd-8*, and NCU03868, are essential for the normal development of protoperithecium or perithecium formation (Kim and Nelson, 2005; Park et al., 2011; Carrillo et al., 2017).

**TABLE 1:** Genes involved in sexual development and melanin synthesis are co-regulated by MAK-2 and MAK-1.

Locus	Function annotation	WT-S/WT-A	Δmak2-S/WT-S	Δmak1-S/WT-S
**Genes involved in sexual development**				
NCU04533	*app*; Normal sexual development (Nowrousian et al., 2007/fungidb).	13.59	0.0015	0.0031
NCU05768	*poi-2*; No protoperithecia and perithecia (Kim and Nelson, 2005).	1,147.27	0.0012	0.0019
NCU04990	*stk-17*, Protein serine/threonine kinase. No protoperithecia and perithecia (Park et al., 2011/fungidb).	2.12	0.4335	0.4757
NCU09915	*fsd-1*, female sexual development-1; No protoperithecia and perithecia (Carrillo et al., 2017/fungidb).	2.95	0.1646	0.41906
NCU06140	*vsd-8*; Reduced protoperithecia and perithecia (Carrillo et al., 2017).	45.72	0.2363	0.1552
NCU03868	Hypothetical protein; Normal protoperithecia. No perithecia and ascospore (fungidb).	9.13	0.2254	0.2007
NCU07743	Named as *protoperithecium formation defect -1*, *pfd-1*; No protoperithecia and perithecia (this study).	38.68	0.1206	0.1581
NCU02250	*oli*, ATP synthase subunit ATP9; No protoperithecia and perithecia (this study).	0.4871	4.19	3.03
NCU05948	Named as *pfd-3*; No protoperithecia and perithecia (this study).	9.85	0.248	0.060
NCU09782	Serine/threonine protein kinase; Very few protoperithecia, no perithecia (this study).	9.55	0.1706	0.3013
NCU09248	*tcf-27*, transcription factor-27; Very few protoperithecia, no perithecia (this study).	3.56	0.2843	0.4336
NCU04930	*cea-6*; Very few protoperithecia, no perithecia (this study).	4.29	0.2188	0.2712
NCU04474	*mol-1*, sulfite oxidase, Very few protoperithecia, no perithecia (this study).	2.72	0.3687	0.3779
NCU00236	*fpo-1*, flavoprotein oxygenase-1; Reduced protoperithecia, no perithecia (this study).	0.3376	8.26	7.41
NCU07776	*acw-5*, anchored cell wall protein 5; Reduced protoperithecia, no perithecia (this study).	0.1487	4.16	4.19
NCU04923	*gcy-1*, Gld1, glycerol dehydrogenase;. Reduced protoperithecia, no perithecia (this study).	3.11	0.2463	0.1874
NCU04936	*gld-1*, glucose dehydrogenase-1; Reduced protoperithecia, no perithecia (this study).	8.87	0.3142	0.4719
NCU08457	*eas*, rodlet protein; Reduced protoperithecia, no perithecia (this study).	0.0882	104.36	10.76
NCU02209	*ods*, fatty acid metabolism-4. Very few perithecia (this study).	11.34	0.2549	0.1234
NCU03422	Named as *reduced perithecium -1* (*rp-1*); Few perithecia (this study).	87.37	0.1089	0.0704
NCU04489	Named as *reduced perithecium -2* (*rp-2*). Few perithecia (this study).	20.41	0.1640	0.2218
NCU09199	*ty-6*, tyrosinase-6; Few perithecia (this study).	33.24	0.0709	0.3957
NCU04952	*gh3-4*, beta-D-glucoside glucohydrolase; Few perithecia (this study).	238.46	0.2071	0.2071
NCU04496	Named as *delayed formation of protoperithecia -1*, *dfp-1*; Delayed formation of protoperithecia (this study).	53.99	0.0286	0.1064
NCU04949	Named as *dfp-2*; Delayed formation of protoperithecia (this study).	9.13	0.2418	0.4044
NCU09976	*ce12-1*, rhamnogalacturonan acetylesterase. Delayed formation of protoperithecia (this study).	9.99	0.2183	0.2007
NCU00830	*tcu-1*, ctr copper transporter; Delayed formation of protoperithecia (this study).	787.87	0.2941	0.2650
NCU07053	*ahd-4*, alhehyde dehydrogenase-4. Delayed formation of protoperithecia (this study).	62.27	0.1937	0.1393
NCU09415	Named as *dfp-3*; Delayed formation of protoperithecia (this study).	235.70	0.4420	0.2378
**Genes participating in melanin synthesis**				
NCU04561	*mld-1*, melanization defective-1; Less melanized perithecia (Carrillo et al., 2017).	232.94	0.0182	0.0198
NCU03584	*per-1, perithecial-1*, polyketide synthase; white perithecia (Ao et al., 2019).	3340.76	0.0767	0.0493
NCU01903	*pkh-1*, polyketide hydrolase; black perithecia (Ao et al., 2019).	7.5838	0.2737	0.2451
NCU05821	*pkh-2*, polyketide hydrolase; black perithecia (Ao et al., 2019).	246.34	0.0141	0.0418
NCU07823	*scy*, scytalone dehydratase; Rust red perithecia, no ascus (this study).	13.64	0.1986	0.5487
NCU06905	*pkr-2/tnr-2/4hnr*, tetrahydroxynaphthalene reductase; Brown-orange perithecia (this study).	64,258	0.0267	0.0249
NCU09390	*pkr-1/tnr-1/3hnr*, tetrahydroxynaphthalene reductase; Brown-orange perithecia, no ascus (this study).	334.89	0.0791	0.0741

*Genotypes with “-A”: samples of N. crassa in vegetative growth stage; Genotypes with “-S”: samples of N. crassa after 3.5 days of sexual development induction. Locus numbers and function were annotated according to the N. crassa genome assembly (http://fungidb.org/fungidb/). RPKM: reads per kilobase of exon model per million mapped reads.*

RNA-seq results showed that the transcript levels of these six genes increased during protoperithecium development in wild type. However, their transcriptional responses to protoperithecium development induction were dramatically impaired in both the Δ*mak*-*2* and Δ*mak*-*1* mutants. After 3.5 days of sexual development induction, the transcript levels of *app*, *poi-2*, *stk-17*, *fsd-1*, and *vsd-8* and NCU03868 in the Δ*mak-2* mutant were only 0.15, 0.12, 43.35, 16.46, 23.63, and 22.54%, respectively, of that in wild type, and the transcript levels of them in the Δ*mak-1* mutant were only 0.31, 0.19, 47.56, 41.9, 15.52, and 20.07%, respectively, of that in wild type (Table 1 and Supplementary Data 2).

### Melanin Synthesis Genes Are Required for Perithecium Development and Downregulated in the Δ*mak-1* and Δ*mak-2* Mutants During Protoperithecium Formation

**FIGURE 3:**
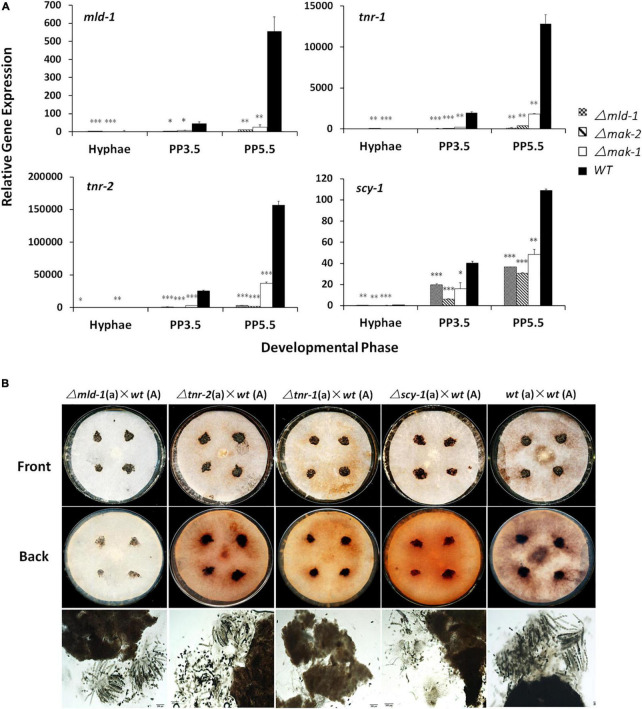
Melanin biosynthetic genes are involved in sexual development of *N*. *crassa*. **(A)** RT-qPCR analysis of *mld*-*1*, *tnr-2*, *tnr-1*, and *scy-1* genes. Hyphae: samples of *N. crassa* in vegetative growth stage; PP3.5: samples of *N. crassa* after 3.5 days of sexual development induction; PP5.5: samples of *N. crassa* after 5.5 days of sexual development induction. Relative gene expression in Δ*mld*-*1*, Δ*mak-1*, and Δ*mak-2* mutants vs. wild-type strain was calculated using 2^– ΔΔ*CT*^ method. All expression levels were normalized to expression of β-tubulin. Bars represent mean and standard error for three replicates. Analysis of variance statistically analyzed differences between mutants and wild type. Values with *P* < 0.001, 0.001 < *P* < 0.01, and 0.01 < *P* < 0.05 are marked with ^***^, ^**^, and *, respectively. **(B)** Perithecium and ascospores production in Δ*mld*-*1*, Δ*tnr-2*, Δ*tnr-1*, and Δ*scy* mutants. Δ*tnr-1* and Δ*scy* mutants cannot form mature asci. Mutants and wild type were used as female parent and first grown on solid crossing medium for 5.5 days under constant darkness at 25°C. Then, a wild-type strain with opposite mating type, as male parent, was inoculated on colony surface of female strains and incubated at 25°C for another 7 days under constant darkness. Perithecia and ascospores formation were checked and imaged.

Melanin is involved in the pigmentation of sexual structures in several fungi (Engh et al., 2007; Ao et al., 2019). According to the RNA-seq results, six of seven melanin synthesis genes, *per-1*, *pkh-1*, *pkh-2*, *pkr-2*/*tnr-2*/*4hnr*, *pkr-1*/*tnr-1*/*3hnr*, and *scy-1*/*sd* (Ao et al., 2019), as well as a transcriptional regulator encoding gene *mld*-*1* that is required for melanin synthesis (Carrillo et al., 2017), were co-regulated by the MAK-1 and MAK-2 proteins (Table 1). To verify these results, *tnr-1*, *tnr-2*, *scy-1*, and *mld-1 genes*, which are involved in the last steps of melanin biosynthesis and its regulation (Carrillo et al., 2017; Ao et al., 2019), were selected for RT-qPCR analysis. As shown in Figure 3A, expression of these four genes was remarkably increased during protoperithecium formation and reached their peak at 5.5 days when protoperithecia became mature and darkened in the wild type. The expression levels of all these genes in both the Δ*mak*-*2* and Δ*mak*-*1* mutants were dramatically lower than those of wild type, verifying that MAK-2 and MAK-1 positively regulate these genes. Moreover, the transcriptional induction of *tnr-2*, *tnr-1*, and *scy-1* during protoperithecium development was dramatically lower in the Δ*mld*-*1* mutant than wild type (Figure 3A). Thus, the MLD-1 protein may function downstream of the MAK-1 and MAK-2 proteins in regulating the melanin synthesis genes.

Interestingly, compared with wild type, the transcript levels of *tnr-2, tnr-1*, *scy-1*, and/or *mld-1* were also lower in the asexual stage in the Δ*mld*-*1*, Δ*mak*-*2*, and Δ*mak*-*1* mutants (Figure 3A), indicating that the basal expression of melanin synthesis genes was also regulated by the MLD-1, MAK-1, and MAK-2 proteins.

The phenotypes of the knockout mutants of the four genes mentioned earlier were also analyzed. All Δ*tnr-2*, Δ*tnr-1*, Δ*scy-1*, and Δ*mld*-*1* mutants can form protoperithecia and perithecia as the female parent. However, the perithecia of the Δ*scy-1* and Δ*tnr-1* mutants were more fragile and easier to disintegrate in the smashing process, and the color of the smashed perithecia is slightly lighter, especially in the Δ*tnr-1* mutant, compared with the dark brown or black in the wild type (Figure 3B). Moreover, due to the secreted melanin, the filter papers covering the SC plates were dark brown after the perithecia of the wild-type strain as the female parent matures. In contrast, the filter papers were brown-orange of the deletion mutants of *tnr-2*, *tnr-1*, or *scy-1* after the perithecia formation or maturation and completely white of the Δ*mld*-*1* mutant, indicating abnormal melanin synthesis and secretion in these mutants during the crossing. More interestingly, we found that the Δ*mld*-*1* and Δ*tnr-2* mutants formed mature asci with eight ascospores, whereas the Δ*tnr-1* and Δ*scy-1* mutants did not. The Δ*tnr-1* mutant cannot detect any mature asci, whereas the Δ*scy-1* mutants can form very few and immature asci with ascospores (Figure 3B). These results indicate that the synthesis of the secreted melanin is not essential for perithecia formation and maturation, but some genes for melanin synthesis are critical for the development of perithecia, asci, and ascospores. Taken together, the melanin synthesis is required for the production of darkened sexual structures, and this process can be co-regulated by the MAK-1 and MAK-2 proteins.

### Mutant Phenotype Analysis of 275 Single-Gene Deletion Mutants of Genes That Were Co-regulated by the MAK-1 and MAK-2 Proteins

Among the 430 genes co-regulated by the MAK-1 and MAK-2 proteins (Figure 1), only 61 have been analyzed for their function in sexual development (Kim and Nelson, 2005; Nowrousian et al., 2007; Fu et al., 2011; Park et al., 2011; Chinnici et al., 2014; Carrillo et al., 2017; Fischer and Glass, 2019).

**FIGURE 4:**
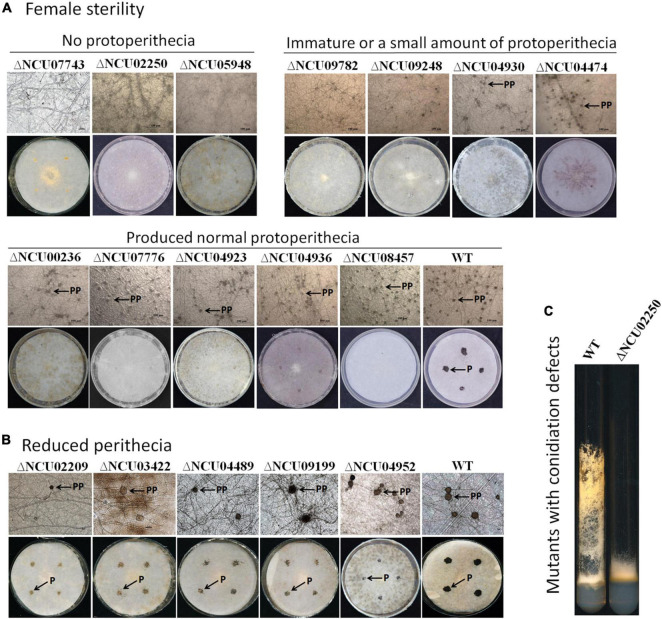
Morphological characteristics of mutants with defects in protoperithecium or perithecium formation. **(A)** Protoperithecium morphogenesis and female fertility. Deletion mutants for indicated genes were used as female parents and inoculated on SC medium covered with cellophane (for observing protoperithecium development) and filter paper (for observing perithecium development), respectively. Plates were incubated for 5.5 days under constant darkness at 25°C. Properithecium development was observed under microscope (40× objective). Then, wild-type strains with mating-type opposite to female strains were inoculated onto female parent colonies grown on plates covered with filter paper. After another 7 days at 25°C under constant darkness, perithecium formation was evaluated and imaged (whole plates). **(B)** Mutants produced fewer perithecia. Protoperithecia were exemplified by arrows with PP; perithecia were exemplified by arrows with P. **(C)** Characteristics of mutants with defects in aerial hyphae. Strains were grown in test tubes containing Vogel’s agar medium at 28°C with continuous light for 7 days.

To do a systematic analysis of the roles played by the MAK-1 and MAK-2 proteins during sexual development, a total of 275 gene deletion mutants were evaluated for their ability to form protoperithecia and perithecia. Two hundred seven of these genes were positively regulated, and 68 genes were negatively regulated by both the MAK-1 and MAK-2 proteins. Mutants of the remaining 94 genes are lethal and/or not available from FGSC. A total of 244 gene deletion mutants showed no defects in protoperithecium and perithecium formation (Supplementary Data 2), but 31 gene deletion mutants showed defects in protoperithecium or perithecium formation to varying degrees (Table 1 and Figure 4). Specifically, single-gene deletion mutants of 12 genes [NCU07743, NCU02250 (*oli*), NCU05948, NCU09782, NCU09248 (*tcf-27*), NCU04930 (*cea-6*), NCU04474 (*mol-1*), NCU00236 (*fpo-1*), NCU07776 (*acw-5*), NCU04923 (*gcy-1*), NCU04936 (*gld-1*), and NCU08457 (*eas*)] displayed female sterility, as no perithecium formed after mating (Table 1 and Figure 4A). Deletion mutants of NCU02209 (*ods*, fatty acid metabolism-4), NCU03422 [named as *reduced perithecia -1* (*rp-1*)], NCU04489 [named as *reduced perithecia -2* (*rp-2*)], NCU09199 (*ty-6*, tyrosinase-6), and NCU04952 (*gh3-4*, beta-D-glucoside glucohydrolase) produced less perithecia as compared with wild type (Table 1 and Figure 4B), and deletion mutants of NCU04496, NCU04949, NCU09976 (*ce12-1*, rhamnogalacturonan acetylesterase), NCU00830 (*tcu-1*, ctr copper transporter), NCU07053 (*ahd-4*, alhehyde dehydrogenase-4), and NCU09415 showed delayed formation of protoperithecia (Table 1).

However, the mutant phenotype of NCU09716, NCU05104, NCU01383, NCU03530, NCU05789, NCU05683, NCU08131, and NCU07180 did not co-segregate well with hygromycin resistance (Chinnici et al., 2014), indicating that additional genetic modifications are likely the cause of the observed deficiencies. Therefore, 12 mutants with female sterility phenotypes can be ascribed to a single gene deletion.

Among the 12 mutants that were female sterile, only deletion mutants for NCU07743, NCU02250 (*oli*), and NCU05948 did not form protoperithecium (Figure 4A, up panel). NCU02250 (*oli*) encodes an ATP synthase subunit ATP9, NCU07743 encodes a TauD domain-containing hypothetical protein, and NCU05948 encodes a hypothetical protein. Therefore, we named NCU07743 and NCU05948 as *protoperithecium formation defect -1* (*pfd-1*) and *pfd-2*, respectively. Interestingly, similar to the △*mak*-*2* and Δ*mak-1* mutants, deletion of NCU02250 led to short aerial hyphae (Figure 4C and Supplementary Figure 1A), suggesting a functional association between the gene NCU02250 and the MAK-1 and MAK-2 kinases.

The remaining nine mutants among the 12 female sterile mutants produced protoperithecia. Deletion mutants of NCU09782, NCU09248 (*tcf-27*), NCU04930 (*cea-6*), and NCU04474 (*mol-1*) produced less protoperithecia (Figure 4A, middle panel), whereas mutants of NCU00236 (*fpo-1*), NCU07776 (*acw-5*), NCU04923 (*gcy-1*), NCU04936 (*gld-1*), and NCU08457 (*eas*) produced a normal amount of protoperithecia (Figure 4A, last panel). These mutants may have defects in protoperithecial maturation, mating, and/or perithecium development, leading to no perithecium formation. Thus, the MAK-1 and MAK-2 proteins can regulate protoperithecial abundance and/or maturation, mating and/or perithecium development.

### Cross-Talk Between MAK-1 and MAK-2 During Sexual Development

As described earlier, our RNA-seq data revealed a large regulatory overlap between MAK-1 and MAK-2, suggesting cross-talk between these two pathways. All three genes in the PR-MAP kinase pathway (*nrc-1*, *mek-2*, and *mak-2*) have increased transcription during protoperithecium development. Specifically, transcription of the *mak-2* gene increased by 7.6-fold in protoperithecia compared with vegetative hyphae (Table 2). For the CWI-MAP kinase pathway, only one gene was upregulated during protoperithecium development. The transcription of the *mak-1* gene in protoperithecia was 2.5-fold greater than in vegetative hyphae (Table 2). Deletion of *mak-2* did not affect the transcription of genes in the CWI-MAP kinase pathway. However, the deletion of *mak-1* affected the expression of the *nrc-1* and *mak-2* genes in the PR-MAP kinase pathway, whose transcript levels after 3.5 days of protoperithecium development induction were only 63 and 52% of that in wild type, respectively (Table 2). These results indicate that the transcriptional response of genes in the PR-MAP kinase pathway to sexual development signal may be regulated by MAK-1.

**TABLE 2:** Transcriptional responses to sexual development induction for 3.5 days by the genes in MAP kinase signal pathway.

Pathway	Kinase	Gene	Locus	Δmak2-A RPKM	Δmak2-S RPKM	Δmak1-A RPKM	Δmak1-S RPKM	WT-A RPKM	WT-S RPKM	WT-S/WT-A	Δmak2-S/WT-S	Δmak1-S/WT-S
CWI	MAPKKK	*mik-1*	NCU02234	1.37	2.11	1.89	1.89	0.64	2.38	3.72	0.89	0.79
	MAPKK	*mek-1*	NCU06419	26.14	52.48	238.37	440.22	41.94	55.58	1.33	0.94	7.92
	MAPK	*mak-1*	NCU09842	39.97	208.48	0	0	42.05	147.83	3.52	1.41	–
PR	MAPKKK	*nrc-1*	NCU06182	14.87	19.44	25.81	28.50	15.54	45.13	2.90	0.43	0.63
	MAPKK	*mek-2*	NCU04612	13.96	54.30	27.26	55.52	16.76	70.98	4.24	0.77	0.78
	MAPK	*mak-2*	NCU02393	0	0	104.56	303.08	68.20	585.69	8.59	–	0.52

*WT, wild type; Δmak2, the mak-2 deletion mutant; Δmak1, the mak-1 deletion mutant; -A, samples of N. crassa in vegetative growth stage; -S, samples of N. crassa after 3.5 days of sexual development induction. Locus numbers and function were annotated according to the N. crassa genome assembly (http://fungidb.org/fungidb/). RPKM, Reads Per Kilobase of exon model per Million mapped reads.*

**FIGURE 5:**
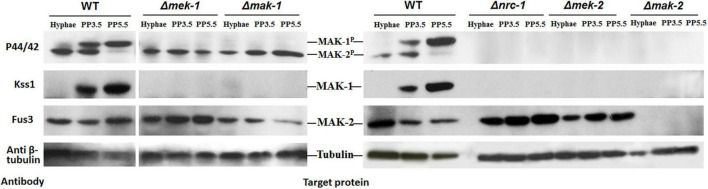
Total protein and phosphorylation status of MAK-1 and MAK-2 in deletion mutants of kinases belonging to PR- and CWI-MAP kinase pathways during protoperithecium morphogenesis. Total protein levels of MAK-1 and MAK-2 were detected by Western blot analysis with antibody Kss1 and Fus3 (Santa Cruz), respectively. Phosphorylation status of MAK-1 and MAK-2 were detected by phospho-p44/42 MAPK antibody (Cell signaling). β-Tubulin was also detected as loading control. Hyphae: samples of *N. crassa* in vegetative growth stage; PP3.5: samples of *N. crassa* after 3.5 days of sexual development induction; PP5.5: samples of *N. crassa* after 5.5 days of sexual development induction.

We next examined the protein levels and the phosphorylation status of the MAK-1 and MAK-2 proteins in all the deletion mutants of kinases (except for *mik*-*1*) belonging to the PR and CWI pathways by Western blotting. In wild-type cells, both the MAK-1 protein and phosphorylated MAK-1 were undetectable in vegetative hyphae but were strongly detected at both 3.5 and 5.5 days after protoperithecium induction (Figure 5). Neither the MAK-1 protein nor phosphorylated MAK-1 was detected in any of the tested MAPK deletion mutants. The MAK-2 protein was detected in both wild-type and mutant (except the Δ*mak-2* mutant) cells at roughly equivalent levels in both vegetative hyphae and protoperithecia. Notably, we observed a slight reduction in the amount of MAK-2 protein in the Δ*mak-1* mutant protoperithecia, which is consistent with our RNA-seq results (Table 2). Phosphorylated MAK-2 was strongly detected in wild-type hyphae and at 3.5 days after protoperithecium induction but was barely detectable at 5.5 days after protoperithecium induction (Figure 5). In contrast, phosphorylated MAK-2 was detected at equivalent levels at every developmental stage in the CWI-MAPK pathway mutants (Figure 5). Phosphorylated MAK-2 was not detected in any of the PR-MAPK pathway mutants.

## Discussion

Sexual development is a complicated process of morphogenesis involving many developmental stages. Here, using comparative transcriptomics as a tool, the regulatory roles and relationships of the PR- and CWI-MAP kinase pathways during protoperithecium development in *N. crassa* were investigated. The transcript levels of all genes in the PR-MAP kinase pathway and *mak-1* gene in the CWI-MAP kinase pathway increased during protoperithecium development, further proving their important roles in sexual development. Also, among the genes whose expression is changed during protoperithecium development, 553 and 699 genes were affected by the deletion of MAP kinase genes *mak*-*1* and *mak*-*2*, respectively. Surprisingly, a total of 430 genes were co-regulated by the MAK-1 and MAK-2 proteins (Figure 1). Thus, protoperithecium formation is regulated by the MAK-1 and MAK-2 proteins. Together with the results of Western blotting, the cross-talk between the MAK-1 and MAK-2 proteins during protoperithecium development was confirmed.

Mutants in different MAP kinase pathways show distinct phenotypes in colony morphology, stress sensitivity, conidiation, and sexual development (Maerz et al., 2008). For sexual development, as revealed by detailed phenotypic analysis, mutants of the MAP kinases were defective at different stages of protoperithecial morphogenesis (Maerz et al., 2008; Lichius et al., 2012). For example, extracellular matrix deposition, hyphal adhesion, and protoperithecium differentiation were absent from mutants of the CWI-MAP kinase pathway, whereas only protoperithecium differentiation and trichogyne emergence were defective in mutants of the PR-MAP kinase pathway (Lichius et al., 2012). All these may suggest that these pathways possess different regulatory mechanisms on protoperithecium development. However, as revealed by our genome-wide transcriptional analysis, 430 genes were co-regulated by the MAK-1 and MAK-2 proteins during early protoperithecium formation, which accounts for 77.8% of the MAK-1 regulated genes and 61.5% of the MAK-2 regulated genes. This means that the regulation of protoperithecium formation by the PR- and CWI-MAP kinase pathways may be similar. This is evidenced by the result that both the Δ*mak*-*1* and Δ*mak*-*2* mutants can form disorganized ascogonial coil and enveloping hyphae during protoperithecium formation (Lichius et al., 2012). The phenotypic difference between the Δ*mak*-*1* and Δ*mak*-*2* mutants may be explained by the difference in regulated genes, as there were still 123 (84 + 39) genes and 269 (221 + 48) genes that were specifically affected by *mak-1* and *mak-2* deletion, respectively (Figure 1). Analysis of these specifically regulated genes may contribute to a more detailed and comprehensive understanding of the molecular mechanism for protoperithecium development under the regulation of these two pathways.

A set of genes are co-regulated by the MAK-1 and MAK-2 proteins, and a large proportion (328 of 430) of these genes are positively regulated by both the MAK-1 and MAK-2 proteins, indicating both kinases mainly exert positive roles in regulating sexual development. This is consistent with the phenotypes of their deletion mutants. Among these genes, many genes are required for sexual development, including previously reported genes (*poi-2*, *stk-17*, *fsd-1*, *vsd-8*, and NCU03863), melanin synthesis genes (*per-1*, *pkh-1*, *pkh-2*, *trn-1*, *trn-2*, *scy-1*, and *mld-1*), and our newly identified 23 genes that are crucial for protoperithecium and perithecium development. The defects in sexual development varied among deletion mutants of these genes. Some were defective in the formation of protoperithecia, whereas some were abnormal in the abundance of protoperithecia, pigmentation, maturation, or mating of protoperithecia, or the timing and abundance of perithecia (Table 1). Thus, the MAK-1 and MAK-2 proteins may regulate different stages of sexual development through different genes, as described earlier. More interestingly, the deletion of NCU02250 led to short aerial hyphae and no protoperithecium formation. Taken together, the MAK-1 and MAK-2 proteins positively regulate sexual development through a set of genes with different functions in protoperithecium and perithecium development.

It is unclear how these genes are co-regulated by the MAK-1 and MAK-2 proteins during protoperithecium development. ADV-1 and PP-1 are the known transcription factors downstream of these two pathways and co-regulate the expression of genes involved in cell wall integrity and cell–cell fusion (Fischer et al., 2018). However, only a small portion of the 430 co-regulated genes would be directly regulated by ADV-1 and PP-1 (Supplementary Data 2). Notably, three transcription factor encoding genes (*fsd-1*, *vsd-8*, and *tcf-27*), which were crucial for protoperithecium development, were found to be regulated by both the MAK-1 and MAK-2 proteins. Deletion of these genes resulted in no or reduced protoperithecium formation. These transcription factors may function downstream of the MAK-1 and MAK-2 signaling pathways involved in the regulation of sexual development. However, no putative phosphorylation sites of MAP kinase were found or predicted in these proteins, indicating they are not the direct target of the MAK-1 and MAK-2 proteins (Jonkers et al., 2014). This is evidenced by the result that *vsd-8* is directly regulated by both ADV-1 and PP-1 (Fischer et al., 2018). However, the direct regulator of *fsd-1* and *tcf-27* is unclear. Thus, in addition to ADV-1 and PP-1, it is possible that at least one transcription factor downstream of the MAK-1 and MAK-2 proteins regulates sexual development through *fsd-1* and *tcf-27*.

The cross-talk between the MAK-1 and MAK-2 proteins is well known in hyphal growth and cell–cell fusion (Fischer and Glass, 2019). Our study extended this cross-talk to the field of sexual development. We found that, during protoperithecium formation, the protein level and phosphorylation status of MAK-1 is dependent on the PR-MAP kinase pathway, whereas the transcriptional and protein level of *mak-2* gene is dependent on the MAK-1 protein. The most interesting thing is that both phosphorylated and total MAK-1 proteins were undetected in all deletion mutants of kinases belonging to the PR-MAP kinase pathway (the Δ*nrc*-*1*, Δ*mek*-*2*, and Δ*mak*-*2* mutants) during protoperithecium development. This is similar to the result of Kamei et al. (2016), in which the phosphorylation of MAK-1 was undetected in the Δ*mak-2* mutant from conidial germination to hyphal development. Recent studies showed that proper fruiting body development in *N*. *crassa* was dependent on the MAK-2-phosphorylated N-terminus of MOB-3, a member protein of the STRIPAK complex, which impacts the nuclear accumulation of MAK-1 (Dettmann et al., 2013). There are also several other proteins HAM-9, HAM-10, AMPH-1, and WHI-2, which affect the activation status of the MAK-1 and MAK-2 proteins during vegetative growth or cell fusion (Fu et al., 2014). Thus, in this study, the abundance and phosphorylation of MAK-1 in a PR-MAP kinase pathway-dependent manner during protoperithecium development may be regulated through a coordinated and complex mechanism, which needs further investigation. Taken together, our study provides an example of functional cross-talk between the PR-MAP kinase pathway and the CWI-MAP kinase pathway during the development of protoperithecia and perithecia.

In summary, this study conducted an in-depth analysis of protoperithecium development by comparative transcriptomics and identified 12 new genes that are essential for female fertility in *N. crassa*. At the same time, the genetic basis and cross-talk between the PR-MAP kinase pathway and the CWI-MAP kinase pathway were revealed, and their downstream gene regulatory networks were elucidated. Our findings shed light on a comprehensive understanding of the genetic basis of the sexual development in *Neurospora* species.

## Data Availability Statement

The datasets presented in this study can be found in online repositories. The names of the repository/repositories and accession number(s) can be found below: https://www.ncbi.nlm.nih.gov/GSE184024.

## Author Contributions

SL and XS designed the study. NL, SY, and WX performed the main experiments. XS, NL, CH, ZC, JH, and SL contributed to the data analysis and the data interpretation. NL, XS, CH, and SL wrote the manuscript. All authors contributed to the article and approved the submitted version.

## Conflict of Interest

JH is employed by Shandong Jinniu Group Company, Ltd., Jinan, China. The remaining authors declare that the research was conducted in the absence of any commercial or financial relationships that could be construed as a potential conflict of interest.

## Publisher’s Note

All claims expressed in this article are solely those of the authors and do not necessarily represent those of their affiliated organizations, or those of the publisher, the editors and the reviewers. Any product that may be evaluated in this article, or claim that may be made by its manufacturer, is not guaranteed or endorsed by the publisher.

## Footnotes

1
www.fgsc.net

2
http://www.fgsc.net/NeurosporaGenomeProject/Sfree/FreeCosegData.htm
