# Supplementary material for: Coordinated Regulation of Protoperithecium Development by MAP Kinases MAK-1 and MAK-2 in Neurospora crassa
Source: Front Microbiol. 2021 Nov 26;12:769615. doi: 10.3389/fmicb.2021.769615 (PMC8662359; doi:10.3389/fmicb.2021.769615)
Supplement: Supplementary file 1 [file Data_Sheet_1.docx]

**Supplementary Figure S1**

**
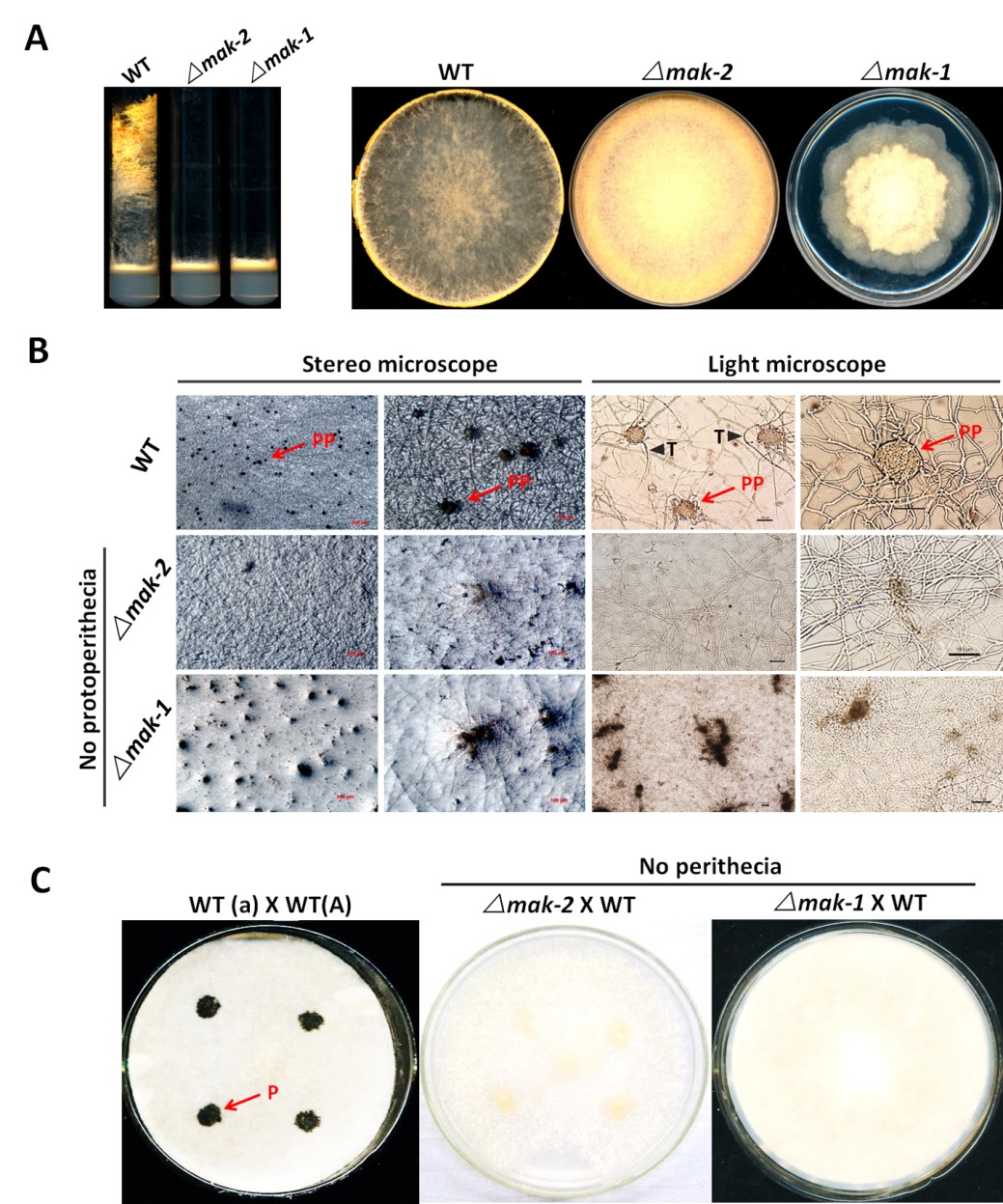
**

**Figure S1.** Morphological characteristics of the *∆mak-1* and *∆mak-2* mutants. A. Asexual colony characteristics of wild type (WT), the *∆mak-1* and *∆mak-2* mutants on solid Vogel’s medium. Strains were grown in test tubes containing Vogel’s agar medium and on Vogel’s plates at 28 ºC with continuous light for 7 days and 5 days, respectively. B. Protoperithecium formation of wild type (WT), the *∆mak-1* and *∆mak-2* mutants. Three microliters of fresh conidial suspension were inoculated on cellophane covered on 0.025 SC medium with 2% sucrose and cultured at 25°C for 7.5 days under constant darkness, and then protoperithecium formation was observed and imaged under stereo and light microscope. Normal protoperithecium were exemplified by arrows labelled with PP, trichogyne were exemplified by triangle labelled with T. C. Perithecium formation detected by crossing test. The mutants and wild type were used as female parent and first grown on solid crossing medium for 5.5 days under constant darkness at 25 ºC. Then a wild-type strain with the opposite mating type, as the male parent, was inoculated on the colony surface and incubated at 25 ºC for another 7 days under constant darkness. Normal perithecia were exemplified by arrows labelled with P.
